# Health assessment of Galapagos pinniped pups during the 2024 El Niño event: hematological, biochemical, and morphometric findings

**DOI:** 10.14202/vetworld.2026.2864-2875

**Published:** 2026-07-10

**Authors:** Eduardo A. Díaz, Andrés Moreira-Mendieta, Carolina Sáenz, Andrea Loyola, Jenifer Suárez-Moncada, Daniel García-Párraga, Pablo Jesús Marín-García, Marjorie Riofrío-Lazo, Diego Páez-Rosas

**Affiliations:** 1 Escuela de Medicina Veterinaria, Colegio de Ciencias de la Salud, Universidad San Francisco de Quito, Cumbaya, Quito, Ecuador.; 2 Fundación GAIAS Europa de la Comunitat Valenciana, Universidad San Francisco de Quito, Valencia, España.; 3 Galapagos Science Center, Universidad San Francisco de Quito, Isla San Cristóbal, Islas Galápagos, Ecuador.; 4 Hospital de Fauna Silvestre Tueri, Instituto iBIOTROP, Universidad San Francisco de Quito, Cumbaya, Quito, Ecuador.; 5 Departamento de Ecosistemas, Dirección Parque Nacional Galápagos, Isla Santa Cruz, Islas Galápagos, Ecuador.; 6 Fundación Oceanogràfic de la Comunitat Valenciana, Ciudad de las Artes y las Ciencias, Valencia, España.; 7 Department of Animal Production and Health, Facultad de Veterinaria, Universidad Cardenal Herrera-CEU, Valencia, España.; 8 Fundación Conservando Galápagos, Galapagos Conservancy, Isla Santa Cruz, Islas Galápagos, Ecuador.

**Keywords:** body condition, climate variability, El Niño–Southern Oscillation, Galapagos fur seal, Galapagos sea lion, hematology, marine mammal health, serum biochemistry

## Abstract

**Background and Aim:**

The Galapagos sea lion (*Zalophus wollebaeki*) and Galapagos fur seal (GFS; *Arctocephalus galapagoensis*) are endemic pinnipeds highly vulnerable to environmental fluctuations associated with the El Niño–Southern Oscillation (ENSO). This study characterized the morphometric, hematological, and biochemical profiles of Galapagos sea lion pups during the 2024 El Niño event and compared them with those of sympatric GFS pups to evaluate species-specific physiological responses to reduced marine productivity.

**Materials and Methods:**

Between March and April 2024, 40 apparently healthy Galapagos sea lion pups from 13 rookeries throughout the Galapagos archipelago were examined. Body weight, standard length, axillary girth, and body condition index were recorded. Blood samples were analyzed for serum biochemical variables, hematocrit, and leukocyte differential counts. Environmental conditions were characterized using Oceanic Niño Index values and satellite-derived chlorophyll-a concentrations. Sex-related and interspecific comparisons were performed using appropriate parametric and non-parametric statistical tests.

**Results:**

No significant sex-related differences were detected in morphometric measurements of Galapagos sea lion pups. Compared with GFS pups, Galapagos sea lion pups exhibited significantly higher body condition indices in both sexes. Female sea lion pups had higher globulin and total protein concentrations and lower albumin and blood urea nitrogen values than female fur seal pups, whereas male sea lion pups showed higher globulin and total protein concentrations but lower glucose concentrations. Most hematological variables overlapped between species, although male sea lion pups exhibited higher hematocrit and lower monocyte and eosinophil counts. During the study period, Oceanic Niño Index values indicated weak-to-moderate El Niño conditions, and chlorophyll-a concentrations were significantly lower than those recorded in 2023, indicating reduced marine productivity.

**Conclusion:**

Galapagos sea lion pups maintained favorable body condition and largely normal clinical profiles despite reduced productivity associated with the 2024 El Niño event. Distinct interspecific differences in body condition and serum proteins suggest species-specific physiological responses to environmental variability. These findings provide valuable baseline health data for long-term monitoring, early detection of nutritional stress, and conservation management of Galapagos pinnipeds under increasingly variable oceanographic conditions.

## INTRODUCTION

Rapid climate change is increasingly affecting marine ecosystems by altering ocean temperature, circulation patterns, and the frequency and intensity of extreme climatic events [1–3]. These changes influence primary productivity, trophic interactions, and prey availability, ultimately affecting the health and survival of upper trophic-level predators [2, 4–6]. Marine mammals are widely regarded as sentinel species because their physiological condition may reflect environmental variability, nutritional stress, disease exposure, and other ecological disturbances [7, 8].

Hematological, biochemical, and morphometric parameters provide objective indicators of physiological status in free-ranging wildlife. Serum biochemical analytes can provide information on metabolic, nutritional, and organ function status, whereas hematological variables, including hematocrit and leukocyte differentials, may help identify inflammatory responses, physiological stress, and changes in immune function [9–11]. Morphometric measurements are also valuable for evaluating growth and body condition. However, baseline clinical data remain limited for many pinniped species, particularly in remote island systems where repeated field sampling is logistically challenging [12, 13]. Expanding these datasets is essential for improving wildlife health assessments and understanding pinniped responses to environmental variability.

The Galapagos archipelago is strongly influenced by the El Niño–Southern Oscillation (ENSO), a major driver of interannual climate variability that interacts with broader global climate change processes [3, 14]. ENSO events modify sea surface temperature, ocean circulation, and nutrient upwelling, often reducing marine primary productivity and altering marine food-web dynamics [4, 15–19]. These changes are especially relevant for endemic otariids such as the Galapagos sea lion (GSL; *Zalophus wollebaeki*) and the Galapagos fur seal (GFS; *Arctocephalus galapagoensis*), whose reproductive success, pup survival, and population trends are closely linked to prey availability. Previous ENSO events have been associated with marked demographic impacts, including population declines and increased pup mortality in both species [20–22].

Recent studies have reported hematological and biochemical reference data for GFS pups under ENSO conditions [23]. However, comparable information for GSL pups remains scarce. This lack of species-specific clinical data limits veterinary interpretation during health assessments and restricts understanding of interspecific physiological responses to environmental stress. Generating such baseline information is particularly important during climatically anomalous periods, when nutritional and physiological challenges may be intensified.

Despite the recognized susceptibility of Galapagos pinnipeds to ENSO-driven environmental fluctuations, information regarding the health status of GSL pups during active ENSO events remains limited. Previous studies have primarily focused on population dynamics, reproductive success, and mortality patterns, whereas comprehensive evaluations that integrate morphometric, hematological, and biochemical parameters remain scarce [20–22]. Furthermore, although recent investigations have established clinical pathology reference data for GFS pups sampled under similar environmental conditions [23], direct interspecific comparisons between sympatric GSL and GFS pups during the same ENSO period have not been adequately investigated. Consequently, species-specific physiological responses to reduced marine productivity remain poorly understood, creating a critical knowledge gap that limits the early detection of nutritional stress and hinders the implementation of effective conservation-oriented health monitoring programs for Galapagos pinnipeds.

Therefore, the present study aimed to characterize the hematological, biochemical, and morphometric profiles of GSL pups during the 2024 El Niño event to assess their physiological status under anomalous oceanographic conditions. Specifically, the study evaluated biochemical indicators of metabolic and organ function, hematological parameters including hematocrit and differential leukocyte counts, and morphometric measurements related to body condition. In addition, the findings obtained for GSL pups were compared with previously reported data from sympatric GFS pups sampled during the same period [23].

By integrating clinical assessments with the prevailing environmental context, this study sought to improve understanding of species-specific physiological responses to reduced marine productivity during an active ENSO event. The findings provide clinically relevant baseline information that may facilitate long-term health monitoring, early detection of nutritional stress, and the development of evidence-based conservation strategies for Galapagos pinnipeds under increasingly variable oceanographic conditions.

## MATERIALS AND METHODS

### Ethical approval

This study was conducted with the authorization and institutional support of Universidad San Francisco de Quito (USFQ) and the Galapagos National Park Directorate (GNPD) under research permits PC-19-23, PC-12-24, and MAAE-DNB-CM-2021-0178-M-0001. All procedures complied with Ecuadorian national regulations governing wildlife research and followed animal care and handling guidelines approved by both institutions. Only free-ranging GSL pups were included in the study, and no animals were maintained in captivity or under human management. Field procedures were designed to minimize handling time, stress, and disturbance to breeding rookeries. Individuals were manually restrained to avoid the risks associated with chemical immobilization. All procedures were performed by USFQ-authorized veterinarians, assisted by trained GNPD park rangers experienced in pinniped handling. Following sample collection, pups were briefly monitored and released at the site of capture.

### Study period and location

**Figure 1:**
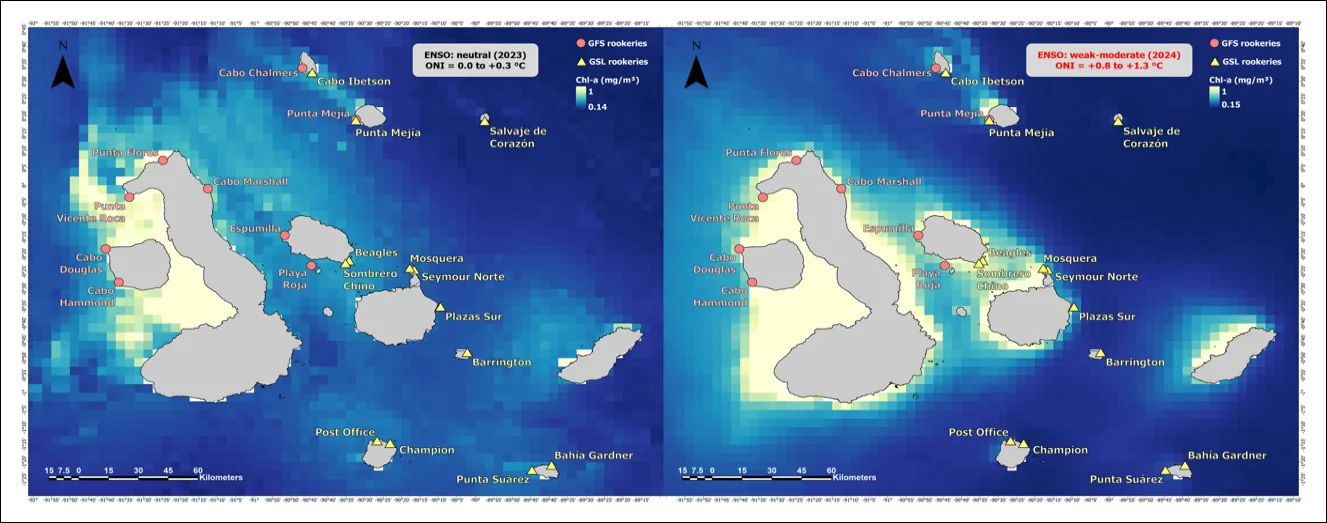
Archipelago-wide spatial distribution of chlorophyll-a (mg m⁻³) in the Galapagos Islands during 2023 (non-El Niño–Southern Oscillation [ENSO] year, left panel) and 2024 (ENSO year, right panel). Oceanic Niño Index values for each year are indicated on the maps. Rookery locations of Galapagos sea lions (yellow triangles) and Galapagos fur seals (red circles) are shown, highlighting site-specific patterns of marine productivity across the archipelago.

The study was conducted during March–April 2024 at multiple rookeries distributed throughout the Galapagos archipelago. A total of 40 GSL pups, representing 10.49% of the pups counted across the sampled rookeries, were examined (Table 1, Figure 1). Environmental conditions during the study coincided with the 2024 El Niño event.

**Table 1:** Sampling locations of GSL pups during March–April 2024, including island, rookery, number of pups sampled, and number of pups counted.

**Sampling date**	**Island**	**Rookery**	**Pups sampled**	**Pups counted**
31 Mar 2024	Pinta	Cabo Ibetson	2	26
01 Apr 2024	Marchena	Punta Mejía	2	2
02 Apr 2024	Genovesa	Salvaje de Corazón	2	37
03 Apr 2024	Rábida	Beagles	5	19
03 Apr 2024	Santiago	Sombrero Chino	2	4
04 Apr 2024	Seymour	Mosquera	5	31
04 Apr 2024	Seymour	Seymour Norte	5	67
05 Apr 2024	Santa Cruz	Plazas Sur	5	61
05 Apr 2024	Santa Fe	Barrington	4	25
06 Apr 2024	Floreana	Champion	2	38
06 Apr 2024	Floreana	Post Office	2	38
07 Apr 2024	Española	Bahía Gardner	2	32
07 Apr 2024	Española	Punta Suárez	2	2
Total	10	13	40	381

GSL = Galapagos sea lion (*Zalophus wollebaeki*).

### Study design

This cross-sectional field study evaluated morphometric, hematological, and biochemical characteristics of free-ranging GSL pups. Pups were identified based on external morphology, particularly the presence of natal fur before molting, which enabled clear differentiation from older juveniles. However, precise chronological age could not be determined. Each pup was captured, handled, and sampled only once. To prevent resampling, individuals were marked with a haircut on the dorsal surface of the hind flippers.

Pups were captured using a hoop net whenever possible during periods of maternal absence to minimize disruption to mother-pup interactions. Capture and manual restraint were performed by trained park rangers, while two wildlife veterinarians simultaneously conducted the procedures to reduce total handling time. To minimize inter-observer variability, the same veterinarian performed all morphometric measurements and blood collections throughout the study. Each pup underwent a brief physical examination and was classified as apparently healthy based on adequate body condition, normal posture and behavior, appropriate responsiveness to handling, and the absence of visible wounds or clinical signs of disease. Sex was determined through external genital examination.

### Morphometric measurements

Morphometric variables included body weight, standard length, and axillary girth, following the same field procedures used in the comparative GFS dataset [23].

Body weight (kg) was measured using a digital scale and net sling (precision ± 0.1 kg), ensuring full support of the animal during weighing. Standard length was measured from the tip of the nose to the end of the tail using a flexible measuring tape to the nearest 0.5 cm. Axillary girth was measured immediately caudal to the fore flippers. Body condition was calculated as body weight (kg) divided by standard length (cm).

### Blood collection

Approximately 5 mL of blood was collected from the caudal gluteal vein using 21-gauge needles. Blood was immediately transferred into ethylenediaminetetraacetic acid (EDTA) tubes (Jiangsu Kangjian Medical Apparatus Co., Ltd., Jiangsu, China) and plain serum tubes (Suzhou Medmay Biotechnology Co., Ltd., Suzhou, China) for hematological and biochemical analyses. Samples were kept in a cooler containing ice packs, transported to a shipboard laboratory, and processed within 4 h of collection. Total handling and sampling time was approximately 10 min per pup to minimize stress and potential handling-related alterations in blood parameters.

### Laboratory analyses

Blood smears were prepared from EDTA-anticoagulated whole blood, air-dried, and stained using a Diff-Quik stain kit (Química Clínica Aplicada S.A., Tarragona, Spain) according to standard hematological procedures [24]. Hematocrit and serum biochemical analyses were performed shortly after sample collection. For serum chemistry, whole blood was centrifuged at 3,500 × g for 10 min to separate serum.

Serum analytes were measured using a portable VetScan VS2 analyzer (Abaxis, Zoetis, Parsippany, NJ, USA) with the VS2 Mammal Profile Plus rotor. The analytes evaluated were alanine aminotransferase (ALT), alkaline phosphatase (ALP), albumin (ALB), globulin (GLOB), total protein (TP), total bilirubin (TB), blood urea nitrogen (BUN), creatinine (CRE), phosphate (PHO), glucose (GLU), and sodium (Na⁺).

Hematocrit was determined using the microhematocrit method by centrifugation of EDTA-anticoagulated blood in capillary tubes at 12,000 × *g* for 5 min, followed by direct reading with a microhematocrit reader. The remaining serum and cellular fractions were stored at −20°C and transported to the USFQ laboratory for potential future analyses.

Differential white blood cell counts were performed 4–6 weeks later at the USFQ laboratory. Stained blood smears were examined under a light microscope (Eclipse Ci-L, Nikon Corporation, Tokyo, Japan) at 1000× magnification using oil immersion. The relative proportions of neutrophils, lymphocytes, monocytes, eosinophils, and basophils were determined by counting 100 leukocytes per smear. All leukocyte differentials were performed by a single trained observer to minimize inter-observer variability.

### Environmental data collection

ENSO conditions during the study period were characterized using the Oceanic Niño Index (ONI) values for seasons overlapping the sampling period. According to the National Oceanic and Atmospheric Administration, the ONI represents the 3-month running mean of sea surface temperature anomalies in the Niño 3.4 region (5°N–5°S, 120°–170°W) [15]. El Niño conditions were classified as ONI > +0.5°C, with weak, moderate, and strong events corresponding to +0.5–0.9°C, +1.0–1.4°C, and ≥ +1.5°C, respectively.

Satellite-derived chlorophyll-a data for the Galapagos archipelago were obtained from the Copernicus Marine Environment Monitoring Service [25] using the “Global Ocean Color Bio-Geo-Chemical, Level-4 monthly and interpolated” product as a proxy for marine primary productivity. Chlorophyll-a concentrations at a spatial resolution of 4 km were extracted for March–April 2023 and March–April 2024. Raster datasets were processed using ArcGIS Pro 3.2 (Esri, Redlands, CA, USA), converted into point features, and mean chlorophyll-a values were calculated within 50-km circular buffers surrounding each rookery. This buffer size was selected to characterize near-rookery marine productivity potentially relevant to local otariid foraging ecology [26, 27]. Each rookery contributed one mean chlorophyll-a value per year.

### Statistical analysis

All analyses were conducted in R version 4.5.1 (R Core Team, Vienna, Austria; https://www.r-project.org/). Descriptive statistics, including mean, standard deviation, and range, were calculated for biochemical, hematological, and morphometric parameters. Normality of each variable was assessed using the Shapiro–Wilk test. Sex-related differences were analyzed using parametric methods for normally distributed data and non-parametric methods for non-normally distributed data. Published reference ranges from related otariid species were used to contextualize the health status of Galapagos pinnipeds.

Descriptive analyses were performed in R version 4.5.1, whereas inferential analyses were conducted using Python 3.11 (Python Software Foundation, Wilmington, USA; https://www.python.org/) with NumPy (NumPy Developers; https://numpy.org/), pandas (The pandas development team; https://pandas.pydata.org/), and SciPy (SciPy Developers; https://scipy.org/) libraries. Statistical significance was set at p < 0.05. Rookery-level mean chlorophyll-a values were evaluated for normality using the Shapiro–Wilk test and for homogeneity of variance using the median-centered Levene's test. Interannual paired differences were analyzed using the Wilcoxon signed-rank test because the data deviated from normality, with each rookery contributing one paired value.

## RESULTS

### Morphometric characteristics of GSL pups according to sex

No significant sex-related differences were detected in GSL pups with respect to body weight, standard length, axillary girth, or body condition (Table 2). Welch’s *t*-tests showed that all comparisons were non-significant (all p ≥ 0.89). Mean differences between males and females were minimal, effect sizes were negligible (Hedges’ *g* ranging from −0.03 to 0.05), and the 95% confidence intervals for all mean differences included zero. Overall, these findings indicate similar morphometric profiles between female and male GSL pups during the sampled postnatal period.

**Table 2:** Morphometric characteristics of Galapagos sea lion pups according to sex.

**Variable**	**Female (n = 26)** ** Mean ± SD**	**Male (n = 14)** ** Mean ± SD**	**Welch's** ***t***	**df**	**p** **-value**	**Mean** **difference (M−F)**	**95% CI** **(M−F)**	**Hedges'** ***g***	**95% CI (** ***g*** **)**
Weight (kg)	13.50 ± 2.49	13.57 ± 2.81	0.08	24.03	0.94	0.07	−1.78 to 1.92	0.03	−0.61 to 0.66
Standard length (cm)	92.66 ± 6.37	92.86 ± 5.96	0.10	28.30	0.92	0.21	−3.94 to 4.35	0.03	−0.60 to 0.67
Axillary girth (cm)	51.06 ± 4.29	50.95 ± 3.78	−0.08	29.86	0.94	−0.11	−2.79 to 2.58	−0.03	−0.66 to 0.61
Body condition (kg/cm)	0.14 ± 0.02	0.15 ± 0.02	0.14	23.01	0.89	0.00	−0.01 to 0.02	0.05	−0.59 to 0.69

Values are presented as mean ± standard deviation. Mean difference (M−F) indicates the difference between males and females. Hedges' *g* represents the standardized effect size corrected for small-sample bias. CI = Confidence interval, F = female, M = male.

### Interspecific comparison of body condition

Body condition was significantly higher in GSL pups than in sympatric GFS pups in both sexes (Table 3). In females, mean body condition was higher in GSL than in GFS, and this difference was significant in both Welch’s *t*-test and Mann–Whitney U test (both p < 0.001), with a large effect size. A similar pattern was observed in males, with significantly higher body condition in GSL than in GFS (both p < 0.001) and a large effect size. These findings indicate consistently higher relative body condition in GSL pups than in GFS pups during the study period.

**Table 3:** Interspecific comparison of body condition between GSL and GFS pups.

**Sex**	**GSL** ** Mean ± SD (n)**	**GFS** **^a^** ** Mean ± SD (n)**	**Mean difference (GSL−GFS)**	**95% CI**	**p-value** **(Welch's ** ***t*** **-test)**	**p-value (Mann–Whitney U test)**	**Hedges' ** ***g***
Females	0.14 ± 0.02 (26)	0.11 ± 0.01 (29)	0.04	0.03 to 0.05	5.44 × 10⁻¹¹	< 0.001	2.54
Males	0.15 ± 0.02 (14)	0.11 ± 0.02 (30)	0.03	0.02 to 0.05	1.70 × 10⁻⁶	< 0.001	1.96

Body condition was calculated as body weight (kg) divided by standard length (cm). Values are presented as mean ± standard deviation. Mean differences are expressed as GSL−GFS. The table also presents 95% CI, p-values obtained from Welch's *t*-test and Mann–Whitney U test, and Hedges' *g* effect sizes. CI = Confidence interval, GFS = Galapagos fur seal, GSL = Galapagos sea lion. Superscript letters indicate literature sources: ^a ^[23].

### Serum chemistry profiles of GSL and GFS pups

Sex-stratified serum chemistry values for GSL and GFS pups are summarized in Table 4. In females, significant interspecific differences were observed for ALB, GLOB, TP, and BUN. ALB concentrations were higher in GFS females than in GSL females, whereas GLOB and TP concentrations were higher in GSL females than in GFS females. BUN concentrations were also higher in GFS females. No significant interspecific differences were detected for ALT, ALP, TB, CRE, PHO, GLU, or Na⁺.

In males, GLOB and TP concentrations were higher in GSL than in GFS, whereas GLU concentrations were higher in GFS than in GSL. No other analytes differed significantly between species in males. Within GSL, females exhibited significantly higher GLOB and TP concentrations than males. No significant sex-related differences were observed within GFS for any serum chemistry analyte.

**Table 4:** Interspecific comparison of serum chemistry analytes in GSL and GFS pups.

**Analyte (unit)**	**GSL Female**	**GFSᵃ Female**	**p-value (species, F)**	**GSL Male**	**GFSᵃ Male**	**p-value (species, M)**	**p-value (sex, GSL)**	**p-value (sex, GFS)**
ALT (U/L)	n = 26; 18.5 ± 6.4; median 19.0; range 8.0–34.0; 95% CI 16.0–21.1	n = 29; 18.1 ± 11.6; median 15.0; range 8.0–64.0; 95% CI 13.7–22.5	0.851	n = 14; 17.6 ± 7.0; median 17.5; range 9.0–36.0; 95% CI 13.6–21.7	n = 30; 16.1 ± 6.8; median 14.0; range 6.0–34.0; 95% CI 13.6–18.7	0.509	0.695	0.441
ALP (U/L)	n = 10; 12.4 ± 9.3; median 10.0; range 2.0–30.0; 95% CI 5.8–19.0	n = 8; 14.5 ± 15.8; median 10.0; range 2.0–51.0; 95% CI 1.3–27.7	0.745	n = 3; 7.7 ± 6.0; median 7.0; range 2.0–14.0; 95% CI −7.3–22.6	n = 16; 13.6 ± 11.7; median 8.5; range 2.0–36.0; 95% CI 7.4–19.8	0.246	0.344	0.884
ALB (g/L)	n = 26; 42.8 ± 6.4; median 43.5; range 26.0–56.0; 95% CI 40.3–45.4	n = 29; 50.4 ± 6.0; median 50.0; range 31.0–60.0; 95% CI 48.1–52.7	<0.001*	n = 14; 45.2 ± 3.5; median 44.5; range 39.0–52.0; 95% CI 43.2–47.3	n = 30; 47.1 ± 7.6; median 49.5; range 23.0–57.0; 95% CI 44.2–49.9	0.275	0.138	0.065
GLOB (g/L)	n = 25; 21.6 ± 6.8; median 20.0; range 11.0–35.0; 95% CI 18.9–24.4	n = 27; 12.0 ± 5.7; median 12.0; range 6.0–36.0; 95% CI 9.7–14.3	<0.001*	n = 14; 17.9 ± 4.1; median 19.5; range 10.0–23.0; 95% CI 15.5–20.3	n = 28; 13.8 ± 7.5; median 13.0; range 5.0–35.0; 95% CI 10.9–16.7	0.027*	0.040*	0.314
TP (g/L)	n = 26; 65.8 ± 5.4; median 65.0; range 57.0–79.0; 95% CI 63.6–68.0	n = 29; 61.5 ± 6.4; median 61.0; range 38.0–73.0; 95% CI 59.0–63.9	0.009*	n = 14; 63.3 ± 2.5; median 63.5; range 57.0–68.0; 95% CI 61.8–64.7	n = 30; 60.8 ± 5.0; median 60.5; range 51.0–69.0; 95% CI 58.9–62.6	0.032*	0.049*	0.635
TB (μmol/L)	n = 25; 6.6 ± 4.3; median 5.1; range 3.4–22.2; 95% CI 4.8–8.3	n = 29; 5.3 ± 1.9; median 5.1; range 3.4–11.9; 95% CI 4.6–6.1	0.186	n = 13; 5.26 ± 2.36; median 5.1; range 3.4–11.9; 95% CI 3.8–6.7	n = 29; 4.9 ± 1.4; median 5.1; range 3.4–8.5; 95% CI 4.4–5.5	0.668	0.235	0.432
BUN (mmol/L)	n = 26; 7.5 ± 4.2; median 6.6; range 2.8–18.2; 95% CI 5.8–9.2	n = 29; 10.4 ± 4.6; median 8.8; range 4.7–20.8; 95% CI 8.6–12.1	0.021*	n = 14; 8.7 ± 5.7; median 7.5; range 2.9–25.6; 95% CI 5.4–12.1	n = 29; 9.0 ± 3.1; median 8.4; range 3.7–16.4; 95% CI 7.8–10.2	0.875	0.499	0.196
CRE (μmol/L)	n = 23; 51.5 ± 22.1; median 44.2; range 17.7–97.3; 95% CI 41.9–61.1	n = 28; 63.5 ± 22.9; median 61.9; range 17.7–132.6; 95% CI 54.6–72.4	0.065	n = 13; 48.9 ± 22.7; median 44.2; range 26.5–114.9; 95% CI 35.2–62.7	n = 30; 60.1 ± 24.5; median 61.9; range 17.7–114.9; 95% CI 50.9–69.2	0.162	0.747	0.593
PHO (mmol/L)	n = 26; 1.6 ± 0.4; median 1.6; range 0.9–2.4; 95% CI 1.4–1.7	n = 29; 1.45 ± 0.38; median 1.39; range 0.90–2.36; 95% CI 1.31–1.60	0.286	n = 14; 1.5 ± 0.4; median 1.5; range 0.7–2.2; 95% CI 1.2–1.7	n = 30; 1.6 ± 0.6; median 1.6; range 0.7–4.3; 95% CI 1.4–1.8	0.416	0.468	0.284
GLU (mmol/L)	n = 25; 6.1 ± 1.3; median 6.1; range 3.1–8.4; 95% CI 5.5–6.6	n = 29; 6.70 ± 1.05; median 6.72; range 4.33–8.60; 95% CI 6.30–7.10	0.067	n = 14; 5.6 ± 1.3; median 5.1; range 4.1–7.7; 95% CI 4.8–6.3	n = 30; 7.2 ± 1.1; median 7.4; range 5.4–9.6; 95% CI 6.8–7.7	<0.001*	0.243	0.069
Na⁺ (mmol/L)	n = 26; 162 ± 4.0; median 162.0; range 154–173; 95% CI 160–164	n = 29; 160 ± 5.0; median 160.0; range 150–170; 95% CI 158–161	0.055	n = 14; 162 ± 7.0; median 162.0; range 151–176; 95% CI 158–166	n = 30; 159 ± 4.0; median 159.0; range 152–167; 95% CI 158–161	0.178	0.969	0.669

Values are presented as n, mean ± SD, median, range (minimum–maximum), and 95% confidence interval for the mean. p-values were calculated using two-sided Welch's *t*-tests, and values marked with an asterisk (*) indicate significant differences (p < 0.05). GFS = Galapagos fur seal, GSL = Galapagos sea lion, ALT = Alanine aminotransferase, ALP = alkaline phosphatase, ALB = albumin, GLOB = globulin, TP = total protein, TB = total bilirubin, BUN = blood urea nitrogen, CRE = creatinine, PHO = phosphate, GLU = glucose, and Na⁺ = sodium. Superscript letters indicate literature sources: ᵃ[23].

### Hematological characteristics of GSL and GFS pups

Hematological values are summarized in Table 5. In females, hematocrit, neutrophils, lymphocytes, monocytes, and eosinophils did not differ significantly between GSL and GFS, whereas basophil percentages were significantly lower in GSL females. In males, hematocrit values were significantly higher in GSL than in GFS, whereas monocyte and eosinophil percentages were significantly lower in GSL males. Neutrophil, lymphocyte, and basophil percentages did not differ significantly between species in males.

**Table 5:** Interspecific comparison of hematological variables in GSL and GFS pups.

**Variable (%)**	**GSL Female**	**GFSᵃ Female**	**p-value (species, F)**	**GSL Male**	**GFSᵃ Male**	**p-value (species, M)**
Hematocrit	n = 23; 41.7 ± 7.3; range 21.0–49.0; 95% CI 38.5–44.8	n = 28; 44.6 ± 6.1; range 32.0–59.0; 95% CI 42.2–46.9	0.133	n = 13; 45.2 ± 3.6; range 40.0–51.0; 95% CI 43.0–47.4	n = 29; 42.1 ± 6.3; range 23.0–49.0; 95% CI 39.7–44.5	0.049
Neutrophils	n = 26; 67.0 ± 9.6; range 46.0–86.0; 95% CI 63.2–70.9	n = 29; 64.4 ± 6.0; range 46.0–73.0; 95% CI 62.1–66.7	0.238	n = 14; 65.0 ± 10.2; range 51.0–85.0; 95% CI 59.1–70.9	n = 29; 66.2 ± 5.3; range 54.0–77.0; 95% CI 64.2–68.2	0.692
Lymphocytes	n = 26; 29.2 ± 9.5; range 11.0–43.0; 95% CI 25.4–33.0	n = 28; 28.9 ± 5.4; range 20.0–41.0; 95% CI 26.8–31.0	0.889	n = 14; 31.4 ± 10.5; range 11.0–48.0; 95% CI 25.3–37.4	n = 29; 26.6 ± 5.0; range 19.0–35.0; 95% CI 24.7–28.5	0.125
Monocytes	n = 26; 2.3 ± 1.0; range 1.0–5.0; 95% CI 2.0–2.7	n = 28; 2.6 ± 2.5; range 0.0–9.0; 95% CI 1.6–3.5	0.658	n = 14; 2.1 ± 0.9; range 1.0–4.0; 95% CI 1.5–2.6	n = 29; 3.3 ± 2.4; range 0.0–10.0; 95% CI 2.4–4.2	0.022
Eosinophils	n = 26; 1.4 ± 2.9; range 0.0–11.0; 95% CI 0.2–2.6	n = 28; 3.0 ± 3.3; range 0.0–11.0; 95% CI 1.7–4.3	0.064	n = 14; 1.4 ± 2.0; range 0.0–6.0; 95% CI 0.3–2.6	n = 29; 3.7 ± 3.5; range 0.0–11.0; 95% CI 2.3–5.0	0.012
Basophils	n = 26; 0.0 ± 0.0; range 0.0–0.0; 95% CI 0.0–0.0	n = 28; 0.4 ± 0.9; range 0.0–4.0; 95% CI 0.1–0.8	0.016	n = 14; 0.1 ± 0.5; range 0.0–2.0; 95% CI −0.2–0.5	n = 29; 0.3 ± 0.7; range 0.0–2.0; 95% CI 0.0–0.6	0.396

Values are presented as n, mean ± SD, range (minimum–maximum), and 95% CI for the mean. *p*-values correspond to interspecific comparisons within each sex using two-sided Welch's *t*-tests. Sample size varied slightly among variables because analyses were performed on a complete-case basis. CI = Confidence interval, GFS = Galapagos fur seal, GSL = Galapagos sea lion. Superscript letters indicate literature sources: ᵃ[23].

### Comparison with published data from other otariid pups

Published serum chemistry ranges for GSL and GFS pups broadly overlapped with those reported for other otariid species for most analytes. The most notable differences were observed for ALP, TB, and Na⁺. Both Galapagos species exhibited lower ALP and higher TB ranges than Antarctic fur seals, South American fur seals, and Steller sea lions, whereas Na⁺ concentrations were generally higher in Galapagos otariids. In contrast, ALT, ALB, TP, BUN, CRE, PHO, and GLU values generally fell within the broad ranges reported for other otariid pups (Supplementary Table S1).

Hematological profiles of GSL and GFS pups also broadly overlapped with published values reported for other otariids. Hematocrit, neutrophil, lymphocyte, monocyte, and eosinophil values were generally comparable across species, whereas basophil values were consistently low or were not reported in the available literature (Supplementary Table S2). 

### Environmental conditions during the study period

**The 2024 sampling period coincided with a weak-to-moderate El Niño event (ONI:** +0.8 to +1.3°C), whereas 2023 represented an ENSO-neutral baseline period (ONI < +0.5°C). Rookery-level chlorophyll-a concentrations were significantly lower in 2024 than in 2023 (Wilcoxon signed-rank test, V = 37, p = 0.005). Across rookeries, the median paired change indicated an overall decline in chlorophyll-a concentrations, although the magnitude of change varied among sites (Supplementary Table S3).

**Figure 2:**
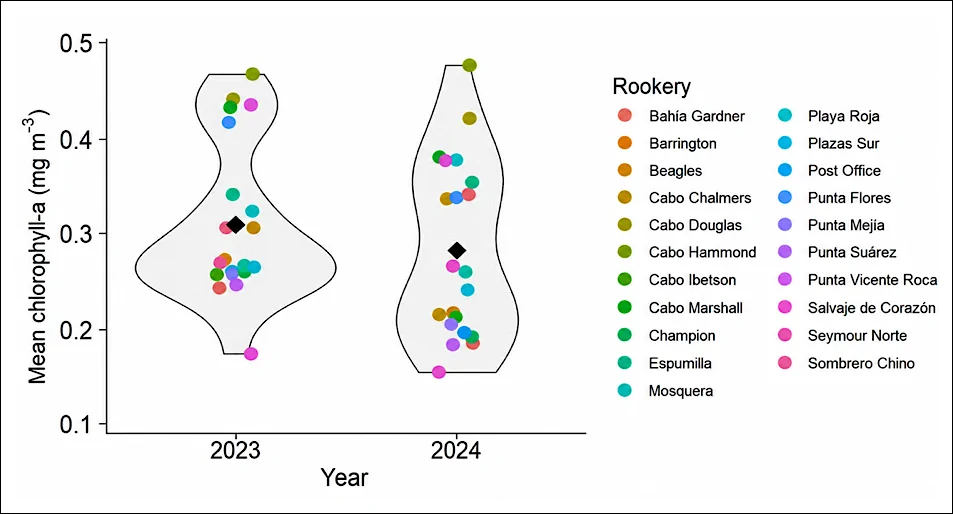
Distribution of rookery-level mean chlorophyll-a concentrations (mg m⁻³) in the Galapagos archipelago during 2023 (non-ENSO year) and 2024 (ENSO year). Violin plots illustrate the density distribution of chlorophyll-a values among pinniped rookeries, with individual rookeries represented by colored points and the archipelago-wide mean for each year indicated by a black diamond.

Despite this general reduction in marine productivity, some local areas retained relatively high chlorophyll-a concentrations, indicating spatial heterogeneity in ENSO-related productivity changes across the archipelago (Figures 1 and 2).

## DISCUSSION

### Physiological status of GSL pups during the 2024 ENSO event

The present study provides an integrated clinical assessment of GSL pups during the 2024 El Niño event and compares these findings with those of sympatric GFS pups using morphometric, biochemical, and hematological parameters. Sampling coincided with a weak-to-moderate ENSO phase and reduced rookery-level chlorophyll-a concentrations relative to 2023, suggesting lower marine productivity during the study period. This environmental context is relevant because Galapagos pinnipeds are known to be sensitive to El Niño-driven oceanographic variability, although the magnitude of ecological effects differs among events [21].

### Morphometric characteristics and body condition

No significant sex-related differences were detected in body weight, standard length, axillary girth, or body condition in GSL pups. These findings indicate morphometric similarity between females and males during early postnatal development and are consistent with previous studies showing that sexual size dimorphism in otariids generally becomes more apparent later in life [28, 29].

Interspecific comparisons revealed clearer differences. Both female and male GSL pups exhibited higher body condition values than GFS pups, indicating better relative body condition during the study period. Body condition indices are widely used as size-adjusted indicators of nutritional status and maternal investment in marine mammals [30, 31]. These interspecific differences may be related, at least in part, to contrasting foraging ecologies. GSLs typically forage in more coastal environments and consume mainly small epipelagic fish, whereas GFSs exploit more pelagic habitats and show a greater contribution of cephalopods to their diet [32]. In addition, GSLs have been reported to exhibit trophic flexibility during ENSO conditions, which may facilitate adjustments in prey use when marine productivity declines [33].

El Niño-associated environmental variability can reduce prey availability and increase maternal foraging effort, with potential downstream effects on pup growth and nursing frequency [20, 34, 35]. However, despite these constraints, the morphometric indicators observed in GSL pups remained relatively stable. This pattern may reflect the moderate intensity of the 2024 ENSO event or short-term physiological resilience, although sampling bias toward apparently healthy individuals should also be considered.

### Serum biochemical profiles

The serum chemistry results revealed a partially similar pattern. GSL pups had higher GLOB and TP concentrations than GFS pups in both sexes, paralleling the interspecific differences observed in body condition. Serum protein levels in marine mammals may vary with nutritional status, hydration, immune activity, and inflammatory processes [10, 11, 36]. Therefore, the higher GLOB and TP concentrations observed in GSL pups may reflect broader interspecific differences in physiological condition, immune stimulation, or both. Nevertheless, because these markers are influenced by multiple biological processes, their interpretation should remain cautious.

In contrast, differences in ALB, BUN, and GLU were restricted to a single sex and should therefore be interpreted conservatively. ALB and BUN concentrations were higher in female GFS pups, whereas GLU concentrations were higher in male GFS pups. Because these analytes can be influenced by recent feeding, fasting duration, hydration, and short-term metabolic variation, such differences may reflect transient physiological states rather than stable species-level patterns [37].

### Hematological characteristics

A similarly cautious interpretation applies to the hematological findings. Most leukocyte differential values overlapped between species, indicating broadly comparable hematological profiles. The few significant differences observed, including higher hematocrit values in GSL males and higher monocyte and eosinophil values in GFS males, may reflect variation in immune maturation, inflammatory status, or parasite exposure rather than consistent interspecific divergence. Previous studies in otariid pups have shown that parasitism and immune development can influence hematological parameters during early life [38, 39].

### Comparison with other otariid species

Comparisons with published data from other otariid pups further support the overall interpretation of the present findings. Most biochemical and hematological analytes fell within ranges previously reported for other otariid species, suggesting that the clinical profiles of both Galapagos pinnipeds remained broadly within expected physiological limits. Nevertheless, ALP and TB showed some divergence from certain published datasets, with both species exhibiting relatively lower ALP and higher TB values. These differences should be interpreted with caution. Variation among studies may reflect differences in age class, nutritional status, health status, sampling context, environmental conditions, or analytical methodology rather than clear biological separation. Although lower ALP and higher TB values could be compatible with differences in metabolic or nutritional status, the present data do not allow a specific causal interpretation [11, 40–43].

### Study limitations

Several limitations should be considered when interpreting this study. First, this was a cross-sectional assessment conducted during a single breeding season, which limits inference regarding temporal variability in physiological parameters. Hematological and biochemical values in marine mammals may vary across seasons, years, and life-history stages according to differences in diet, development, and metabolic demands.

Second, sample size limitations are inherent to studies of free-ranging marine mammals, particularly in remote and protected environments such as the Galapagos archipelago. Logistical constraints, animal welfare considerations, and restricted access to rookeries can limit the number of animals sampled and reduce statistical power.

Third, the study focused on apparently healthy pups accessible within breeding rookeries. Consequently, the reported values likely represent relatively healthy individuals and may underestimate the full range of health variability within the population.

Fourth, interpretation of clinical pathology in wildlife is constrained by the limited availability of species-specific reference intervals and methodological differences among studies. Variability in analytical platforms, laboratory procedures, and sample handling may influence reported values and complicate direct interstudy comparisons.

Fifth, capture and restraint may have influenced some blood analytes, particularly GLU concentrations and leukocyte-related variables, despite efforts to minimize handling time and disturbance.

### Implications for conservation and health monitoring

Despite these limitations, this study provides the first direct comparison of GSL and GFS pup health during an active ENSO event. GSL pups exhibited higher relative body condition and higher GLOB and TP concentrations than GFS pups, whereas most hematological variables were similar between species. These findings suggest species-specific differences in physiological responses to reduced marine productivity, while also indicating broadly comparable clinical status across most measured parameters.

The baseline data generated in this study provide a useful foundation for long-term monitoring, early detection of nutritional stress, and conservation-oriented health assessment of Galapagos pinnipeds under increasingly variable oceanographic conditions.

### Future perspectives

The present study evaluated morphometric and standard clinical pathology parameters but did not assess pathogen exposure, endocrine responses, or chronic stress biomarkers. Future studies integrating parasitological, immunological, and endocrine markers, together with longitudinal monitoring during both ENSO and non-ENSO periods, would provide a more comprehensive understanding of pinniped health under changing oceanographic conditions.

## CONCLUSION

This study provides the first integrated comparison of the morphometric, biochemical, and hematological characteristics of GSL and sympatric GFS pups during an active ENSO event. Despite reduced marine productivity associated with the weak-to-moderate 2024 El Niño conditions, GSL pups maintained favorable body condition and exhibited clinical profiles that largely fell within the physiological ranges reported for other otariid species. Interspecific comparisons revealed consistently higher body condition, GLOB, and TP concentrations in GSL pups, whereas most hematological variables showed broad overlap between species, suggesting largely comparable health status with some species-specific physiological differences.

A major strength of this study lies in its integrated approach, combining morphometric measurements with serum biochemical and hematological assessments under naturally occurring ENSO conditions. Furthermore, the direct comparison of sympatric GSL and GFS pups sampled during the same period provides valuable insights into species-specific responses to reduced marine productivity and establishes clinically relevant baseline information for both species. The incorporation of environmental indicators, including ONI and chlorophyll-a concentrations, further strengthens the ecological interpretation of the findings.

Overall, the results indicate that GSL pups exhibited apparent physiological resilience during the 2024 El Niño event despite evidence of reduced ocean productivity. The baseline data generated in this study contribute to a better understanding of the health status of Galapagos pinnipeds and provide an important foundation for long-term health surveillance and conservation programs. Continued multidisciplinary monitoring across ENSO and non-ENSO periods will be essential for detecting early signs of nutritional or physiological stress and for improving conservation strategies for these endemic marine predators in the face of increasing environmental variability and climate change.

## DATA AVAILABILITY

The supplementary data can be made available from the corresponding author upon request.

## GENERATIVE AI DECLARATION

The authors declare that no generative artificial intelligence (AI) or AI-assisted technologies were used in the writing, analysis, or preparation of this manuscript.

## AUTHORS’ CONTRIBUTIONS

ED, MRL, and DPR: Conceptualization. ED, AMM, DGP, MRL, and DPR: Investigation. ED, AMM, CS, AL, JSM, PMG, and MRL: Methodology. ED, AMM, and CS: Formal analysis. MRL and DPR: Project administration and supervision. ED and DPR: Writing – original draft. ED, AMM, CS, AL, JSM, DGP, PMG, MRL, and DPR: Writing – review and editing. All authors have read and approved the final manuscript.
